# *Twilight* reloaded: the peptide experience

**DOI:** 10.1107/S205979831601620X

**Published:** 2017-02-28

**Authors:** Christian X. Weichenberger, Edwin Pozharski, Bernhard Rupp

**Affiliations:** a k.k. Hofkristallamt, 991 Audrey Place, Vista, CA 92084, USA; b Department of Biochemistry and Molecular Biology, University of Maryland School of Medicine, Baltimore, Maryland, USA; c Department of Genetic Epidemiology, Medical University Innsbruck, Schöpfstrasse 41, A-6020 Innsbruck, Austria

**Keywords:** protein–peptide ligand structures, validation, evidence-based reasoning, OMIT difference density

## Abstract

The *de facto* commoditization of biomolecular crystallography as a result of almost disruptive instrumentation automation and continuing improvement of software allows any sensibly trained structural biologist to conduct crystallo­graphic studies of biomolecules with reasonably valid outcomes: that is, models based on properly interpreted electron density. Robust validation has led to major mistakes in the protein part of structure models becoming rare, but some depositions of protein–peptide complex structure models, which generally carry significant interest to the scientific community, still contain erroneous models of the bound peptide ligand. Here, the protein small-molecule ligand validation tool *Twilight* is updated to include peptide ligands. (i) The primary technical reasons and potential human factors leading to problems in ligand structure models are presented; (ii) a new method used to score peptide-ligand models is presented; (iii) a few instructive and specific examples, including an electron-density-based analysis of peptide-ligand structures that do not contain any ligands, are discussed in detail; (iv) means to avoid such mistakes and the implications for database integrity are discussed and (v) some suggestions as to how journal editors could help to expunge errors from the Protein Data Bank are provided.

## Abstract 2

The potential causes of the misinterpretation of peptide density in a significant number of protein–peptide complex structures are analyzed, together with suggestions for good practice and specific education aimed at minimizing overinterpretation and mistakes in protein–peptide complex structure models.

## 1. Introduction  

Falsehood flies, and truth comes limping after it, so that when men come to be undeceived, it is too late; the jest is over, and the tale hath had its effect. (Jonathan Swift, 1667–1745).

Biomolecular crystallography enjoys a rigour of method that is rarely found in other branches of biomedical science. Macromolecular crystallographers have been at the forefront of comprehensive data and model deposition, and most journals have followed suit in insisting on the mandatory deposition of model coordinates and diffraction data. The process of converting raw data to processed diffraction data sets as deposited in the Protein Data Bank (PDB) (Berman, 2008 ▸), and from there to the resulting electron-density reconstruction, is of respectable mathematical objectivity. In contrast, the interpretation of this electron-density reconstruction in terms of an atomic model allows not insignificant individual freedom (Kleywegt & Jones, 1995 ▸), which in turn can be affected by various cognitive biases, collectively termed ‘wishful thinking’. Such cognitive biases have been recognized since the early Enlightenment (Bacon, 1620 ▸), and have been put in the context of crystallo­graphic model interpretation, for example, by Rupp (2008 ▸), Pozharski *et al.* (2013 ▸) and Dauter *et al.* (2014 ▸).

In the ‘standard model’ of epistemological empirical reasoning, the model likelihood is dependent on the degree of evidence provided for the particular model or hypothesis, weighted by the probability of the hypothesis or model being compatible with independently gained prior knowledge (Bayes, 1763 ▸). In its modern form, this basic epistemology of inductive reasoning was formulated by Laplace (1814 ▸), and we quote from an English translation (Truscot & Emory, 1902 ▸):

…the more extraordinary the event, the greater the need of its being supported by strong proofs. For those who attest it, being able to deceive or to have been deceived, these two causes are as much more probable as the reality of the event is less.

We take as self-evident that ‘The proposition that a ligand in a complex structure is in a specific position and exhibits a unique conformation (*i.e.* is present in a specific pose) is a very strong and powerful statement, and accepted scientific epistemology requires that strong claims are backed by correspondingly strong evidence’ (Pozharski *et al.*, 2013 ▸) also holds for peptide ligands.

### 1.1. Small-molecule ligands *versus* peptide ligands   

One of the difficulties that affect the assessment of small-molecule ligand structures originates from the fact that while the judgment of evidence in terms of the quality of the fit to the electron density can be determined in practice with reasonable reliability (Kleywegt *et al.*, 2004 ▸; Tickle, 2012 ▸), the specifics of the *a priori* expected stereochemistry can be highly context-sensitive. Bond lengths and distances can be very accurate, but only if the actual chemical state of the ligand, including tautomerization, chemical modifications and correct stereochemistry (Bax *et al.*, 2017 ▸), is known. Small-molecule conformational energies can also be evaluated using quantum mechanics (Beshnova *et al.*, 2017 ▸) or empirical searches (Emsley, 2017 ▸), but in general the freedom of conformation of small-molecule ligands is quite high. For this reason, in our previous small-molecule ligand compilation, *Twilight* (Pozharski *et al.*, 2013 ▸; Weichenberger *et al.*, 2013 ▸), we abstained from rendering any judgement about prior stereochemical probabilities, which find their manifestations in the so-called (stereochemical) restraint files (Long *et al.*, 2017 ▸; Moriarty *et al.*, 2017 ▸).

The situation regarding the use of restraints for validation is decidedly different for peptide ligands bound to a protein target. For standard amino-acid residues, peptide geometry is well established and fairly predictable, and peptide-backbone torsion angles (Ramachandran & Sasisekharan, 1968 ▸) are normally not restrained. The backbone torsion angles therefore provide *de facto* a geometric cross-validation set of the bound peptide geometry. If there is insufficient electron density for the peptide, then it is very likely that the refinement will lead to unreasonable peptide-backbone geometry; that is, implausibly high-energy backbone conformations which are readily detectable as Ramachandran outliers (Kleywegt & Jones, 1996 ▸; Lovell *et al.*, 2003 ▸).

Compared with small-molecule ligands, the availability of two powerful and independent local validation criteria, the evidence-based real-space fit (Brändén & Jones, 1990 ▸; Kleywegt *et al.*, 2004 ▸; Rupp, 2006 ▸; Tickle, 2012 ▸) and the prior probability-based backbone-geometry plausibility, allows the straightforward detection of models with vanishing posterior likelihood. A bad density fit of a peptide ligand almost always leads to poor backbone geometry; taken together, these two measures allow a highly accurate assessment of problems with the model.

The peptide-ligand quality scores can be further enhanced by including standard geometry measures, such as bond lengths and bond angles (which however are generally fairly restrained during refinement) and torsion-angle outliers (which are subject to weaker restraints and generally require interpretable electron density to be specified), and the *B*-factor differences between the peptide ligand and nearby protein target residues (*cf.* §[Sec sec3.3]3.3).

## 2. Technical and cognitive challenges in ligand structure modelling   

In our previous *Twilight* publication (Pozharski *et al.*, 2013 ▸), we discussed at some length the major technical and cognitive difficulties encountered in modelling ligands. With some additions, the same holds for peptide ligands and we only briefly summarize the challenges here, with reference to the corresponding sections in Pozharski *et al.* (2013 ▸). A recent compilation and review of most validation tools for protein–ligand structures is provided in Deller & Rupp (2015 ▸). Generic recommendations for best practices for the deposition and validation of ligand structure models proposed at the First wwPDB/CCDC/D3R Ligand Validation Workshop (30–31 July 2015) have been published (Adams *et al.*, 2016 ▸).

### 2.1. Global reciprocal-space statistics are insensitive to local errors   

Each atom *j* at a given position **x** *_j_* in the unit cell of the crystal contributes to each structure factor **F_h_** and therefore to *each* observed reflection intensity *I* **_h_**, from which the structure-factor amplitudes *F*_obs_ (*F*_o_) for each reflection **h** are computed, 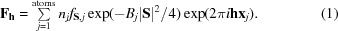



#### 2.1.1. Low scattering mass   

The contribution of each atom to **F_h_** is, according to (1), proportional to its electron count (expressed as the angle-dependent scattering factor *f*_**S**,*j*_) weighted by the occupancy *n_j_* and the exponential *B*-factor term, which in essence is a measure of the probability of finding the atom at its stated position **x** *_j_*. The scattering mass of a ligand molecule of several hundred daltons for small molecules, up to a few thousand daltons for complete peptides, is small compared with the target protein mass of perhaps several tens to hundreds of kilodaltons, and therefore the ligand contribution to the total scattering is often two to four orders of magnitude less than that of the protein partner.

#### 2.1.2. Incomplete ligand binding   

The already small ligand scattering contribution is frequently further diminished by low ligand occupancies, which can only asymptotically reach 100% in optimal cases of tight binding (see Fig. 1 in Pozharski *et al.*, 2013 ▸). In addition, binding sites have by nature evolved to attract ligand moieties. While specifics assure that the correct substrate is processed *in vivo*, even remotely similar molecular moieties (*i.e.* anything from expression-host cellular contents to purification buffers to crystallization-cocktail components) can be forced by high concentration into a binding site (and can even partly replace or entirely compete out the desired ligand). Such molecular stowaways can insidiously produce some kind of obscure partial electron density in the binding site that beckons to be filled with a desired ligand or peptide.

#### 2.1.3. Enhanced mobility   

As a result of conformational flexibility or dynamics of the ligand, the displacement measure *B_j_* of the atoms increases and the associated negative exponential term (‘temperature factor’) reduces the scattering contribution further. The conformational freedom of a ligand frequently increases with distance from the specifically bound parts of the ligand. The latter is particularly problematic in the case of large ligands and even more so in the case of extended peptide chains. The situation is similar in the case of glycan decorations, which often become untraceable with increasing length of the polysaccharide branches (see the examples in Pozharski *et al.*, 2013 ▸).

The consequence of the above is that *absolute* values of global reciprocal-space quality measures based on the linear residual between observed *F*_obs_ (*F*_o_) and calculated structure-factor amplitudes *F*_calc_ (*F*_c_), *i.e.* the *R* values, cannot clearly indicate whether a ligand is present or not, nor whether such a ligand has been properly placed, modelled or refined. In addition, as a global measure, *R* values cannot pinpoint where a specific error in a model might be located.

### 2.2. Local real-space quality measures are not failsafe either   

In contrast to *global reciprocal-space metrics* such as *R* values or coordinate precision estimates (Cruickshank, 1999 ▸), *local real-space metrics* in principle allow specific errors in a model to be pinpointed. However, some distinctions and caveats are necessary.

#### 2.2.1. Evidence-based real-space measures   

These measures are applied to the direct evidence term; that is, they measure the fit of the model to the electron density. They are evaluated locally, with an ultimate granularity of a single atom for the real-space *R* value (RSR) or real-space correlation coefficient (RSCC). However, these measures can be averaged, with the benefit of providing a more compact statistic, but at the cost of losing detail. For example, the local ligand density fit (LLDF; Read *et al.*, 2011 ▸) listed in the PDB validation reports is computed for the entire ligand, with no possibility of discerning well modelled parts of a larger ligand from more conjectural pieces. The question arising of how, and to what degree, to model those parts of a peptide or ligand that are absent in electron density will be discussed in §[Sec sec5]5.

#### 2.2.2. Prior knowledge-based real-space measures   

Measures to judge to what degree the model fulfils prior expectations are again applicable at high granularity, but can be averaged at the cost of losing local detail. Typical examples are stereochemical parameters, which are often known with high certainty, such as bond lengths, bond angles, chirality or torsion-angle preferences. Caveats for relying on them as sole validation criteria are that (i) owing to the general poor determinacy of macromolecular refinement, the prior expectations introduced as stereochemical restraints effectively act as data points in macromolecular posterior maximum-likelihood refinement. As a consequence, the fewer data that are available the more the model relies on, and therefore *reflects*, these prior expectations (as an extreme instance, a purely computational model without any experimental data support may have perfect stereochemistry). As soon as sufficient data are available and restraints can be relaxed, outliers from expectation values become more and more indicative of either actual errors, or if evidence in the form of electron density supports them, of interesting high-energy (hot-spot) features in the structure. The second problem, as explained in §[Sec sec1]1, is that (ii) when the prior expectations are wrong (most frequently manifested as an incorrect restraint file or different actual ligand stereochemistry or chemical composition), false error flags result.

Peptide ligands benefit in the case of caveat (i) from the fact that backbone torsion angles are generally not restrained in refinement and therefore act as a geometric cross-validation that is insensitive to artificial restraint. For caveat (ii), expectation values for peptide stereochemistry are generally well established and peptides in general exhibit more predictable conformational variation compared with complex small-molecule ligands.

### 2.3. Electron-density interpretation   

A protein structure model is the end result of repeated cycles of model building and refinement, and the electron density is the primary crystallographic evidence for the presence and location of the model atoms. The fit of the model to minimally biased electron density is therefore also the primary indicator of local model quality, including peptide ligands. Various statistical measures exist to quantify and visualize the correspondence between model and electron density, primarily the RSR and RSCC, as well as difference density measures. These were introduced decades ago (Brändén & Jones, 1990 ▸), their usefulness has repeatedly been reiterated (see, for example, Rupp, 2006 ▸), and they are publicly available for deposited PDB structures through the Uppsala Electron Density Server (EDS; Kleywegt *et al.*, 2004 ▸), *PDB_REDO* (Joosten *et al.*, 2011 ▸) or the PDBe at the EBI (Velankar *et al.*, 2016 ▸). Despite their undisputed practicality for real-space model validation, both the RSR and the RSCC have the flaw of not distinguishing between model accuracy and model precision. More sophisticated statistical measures for real-space validation (Tickle, 2012 ▸) can distinguish between local model accuracy and model precision (with the latter ultimately depending on the data quality). Real-space validation scores and other quality indicators have been summarized in Weiss & Einspahr (2011 ▸).

The electron density is commonly presented in the form of σ_A_-derived maximum-likelihood (ML) maps (Read, 1986 ▸; Pannu & Read, 1996 ▸) with Fourier coefficients of the form (2*mF*_o_ − *DF*_c_)exp(*i*φ) suitable for initial building, and difference density maps of the form (*mF*_o_ − *DF*_c_)exp(*i*φ) best suited for model correction. Here, *m* is the figure of merit (directly related to the mean phase angle uncertainty as *m* = 〈cosΔφ〉), *D* is the Luzzati factor (Luzzati, 1953 ▸) and φ is the phase angle calculated from the model. The derivation and meaning of the ML coefficients are summarized, for example, by Rupp (2009 ▸).

#### 2.3.1. Proper use of difference electron-density maps   

An *F*_o_ − *F*_c_-type difference map of a protein–peptide complex structure that actually contains a peptide ligand will show distinct positive difference density for the omitted ligand (Bhat, 1988 ▸; Terwilliger *et al.*, 2008 ▸). A difference map calculated with a placed ligand where no ligand exists will show equally distinct negative difference density for the ligand. It is therefore imperative to declare in electron-density figures the type of (difference) density that is being displayed, the contour level and the procedure through which the electron density was generated. Difference density analysis is meaningful only when either (i) the model contributes to the calculated scattering factors *F*_c_ and/or (ii) there is an actual experimental contribution of a ligand in the observed structure factors *F*_o_. If the ligand model does not contribute to *F*_c_ *and* the *F*_o_ data do not contain any ligand contribution, then a Fourier synthesis based on the *F*_o_ − *F*_c_ difference becomes a noise-level subtraction and is meaningless (see Table 1 in Pozharski *et al.*, 2013 ▸). This is the case with either extreme *B* factors refined for an absent peptide ligand and/or with very low (partial) ligand occupancies.

#### 2.3.2. Contouring of electron-density maps   

It may be tempting to contour the electron density down until some noise features start to appear in a binding site, ready to be misinterpreted and modelled as the desired peptide. Occasionally a reasonable-appearing 2*mF*_o_ − *DF*_c_ density figure may even be produced, but the absence of clear *positive OMIT difference density* will serve well as real-space cross-validation. Noise density levels are reached in normal 2*mF*_o_ − *DF*_c_ maps below approximately 0.7σ, but in very clear (OMIT) maps of quality models obtained from excellent data inspection at lower levels may be justifiable. The hazard of improperly contouring down to noise levels and then displaying the electron density only within the surroundings of the atomic model has been pointed out repeatedly (Rupp & Segelke, 2001 ▸; Stanfield *et al.*, 2016*a* ▸,*b* ▸; Rupp, 2016 ▸). While at first glance visually plausible 2*mF*_o_ − *DF*_c_ maps can be generated, a post-publication examination will almost always reveal the biased and potentially misleading presentation.

Interpretation of difference maps (*mF*_o_ − *DF*_c_) for a nearly final model is more rigid. With almost all of the ordered structural elements and bulk solvent accounted for, variation in the remaining electron density simply reflects the noise level in the underlying data. In practice, the >3σ level is generally accepted as above noise, in part because it is the default contouring level in the popular display program *Coot* (Emsley *et al.*, 2010 ▸) as the initial point in difference map inspection. The recommendations for OMIT difference electron-density map reconstruction, contouring and general inspection of maps described in *Twilight* (Pozharski *et al.*, 2013 ▸) remain the same for peptide ligands.

### 2.4. Human factors: the peptides of desire   

As introduced in §[Sec sec1]1, accepted scientific epistemology requires the practitioners of crystallography to assess their beliefs and expectations in view of the (sometimes painful) necessity of balancing experimental evidence against desired outcomes (see, for example, Rupp, 2010 ▸). Cognitive biases which create the tendency to find what one seeks (Bacon, 1620 ▸) and to ignore contradictory evidence (or the absence of evidence) are well documented in the psychological science literature and are known as expectation bias and confirmation bias, respectively (Koehler, 1993 ▸; Simmons *et al.*, 2011 ▸). Models of proteins complexed with small-molecule or peptide ligands usually carry exciting fundamental information such as elucidating the mechanism of an enzymatic reaction, or they provide high-impact, sometimes commercially valuable, information about drug–target or antigen–antibody inter­actions. This potential of significant intellectual and pecuniary rewards carries with it a responsibility to ensure that the asserted claim is sound by providing a valid protein–ligand structure model that is supported by crystallographic evidence; that is, distinct positive OMIT electron density for the entire modelled ligand.

## 3. Methods   

### 3.1. Data mining   

We used the PDB Online Advanced Search interface to retrieve a list of 55 741 entries present in the database as of 6 April 2016 which were determined by X-ray crystallography and which contained at least two peptide chains without any further DNA or RNA chains. For any multimer in such PDB entries, a ‘peptide-ligand chain’ was defined as a peptide chain with fewer than 50 residues and shorter than a fifth of the length of the longest peptide chain of the multimer. Applying this definition and dropping those entries without electron-density information (Kleywegt *et al.*, 2004 ▸) resulted in 9805 peptide-ligand chains distributed across 5667 PDB entries.

In our analysis of peptide-ligand chains, we made use of several different protein structure quality indices introduced in §[Sec sec1]1 and combined them into a single score by applying a likelihood function. More specifically, we incorporated geometric measures [derived from the numbers of (i) bond-length outliers, (ii) bond-angle outliers, (iii) outliers from the Ramachandran allowed region and (iv) protein side-chain rotamer outliers], electron-density model quality scores based on the real-space correlation coefficient and real-space *R* values, and finally a score based on the *B* factor of the atoms from the peptide-ligand chain compared with neighbouring atoms. All data were retrieved from wwPDB X-ray structure-validation report XML files (Velankar *et al.*, 2016 ▸) except for atomic *B* factors, which were extracted in batch mode with *PyMOL* (DeLano, 2008 ▸).

### 3.2. Geometric measures   

The geometric descriptors we extracted from the wwPDB validation report were calculated with the *MolProbity* suite (Chen *et al.*, 2010 ▸). For each chain, we obtained the number of Ramachandran outliers; that is, the number of residues with φ/ψ backbone angles that are neither in the favored nor in the allowed region of the *MolProbity* Ramachandran plot. Furthermore, we counted the number of residues that had a nonrotameric side-chain conformation. Following the recommendations of the wwPDB X-ray validation task force (Read *et al.*, 2011 ▸), we counted the number of bond lengths and bond angles that differed by more than five standard deviations from their expected values. Dividing the counts by the appropriate residue number resulted in a chain-length-independent measure.

### 3.3. *B*-factor comparison to neighbouring atoms   

When a peptide ligand is fitted poorly into electron density or lacks supporting electron density, the refinement program tries to reduce the scattering contribution by increasing the *B* factors of the offending atoms. The situation is then characterized by a distinctive rise in atomic *B* factors when compared with spatially neighbouring protein atoms that do not belong to the poorly fitted peptide-ligand chain. We ran *PyMOL* to query occupancy-weighted *B* factors of non-H atoms for both the peptide-ligand chain and for any atoms within a 6 Å neighbourhood of this chain. A simple difference score to express the relationship of *B* factors between the peptide-ligand chain and its environment was calculated by subtracting the average *B* factor of the neighbouring atoms from the average *B* factor of the peptide-ligand chain. For any such *B*-factor average difference, we computed its percentile based on the empirical difference score distribution *B* of all chains investigated.

### 3.4. Electron-density-based measures   

**Figure 1:**
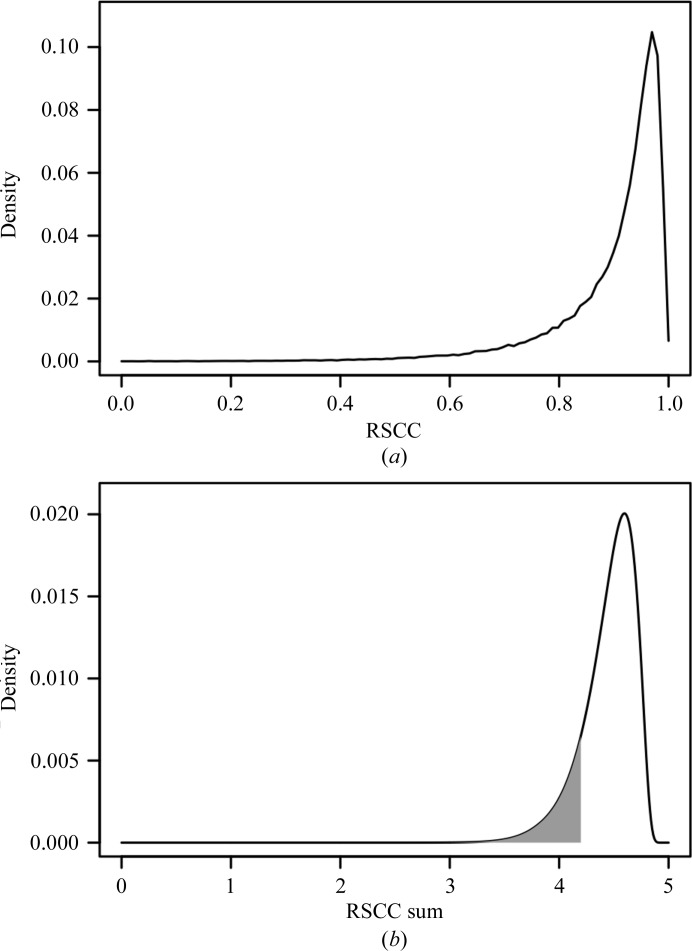
Probability density functions of RSCC values and sums. (*a*) Graph of *f*_1_, the empirical p.d.f. of RSCC values derived from 112 565 residues belonging to peptide-ligand chains, with the bin size set to 0.01. (*b*) Graph of *f*_5_, which is the p.d.f. of RSCC sums *S*_5_, calculated by iterative convolutions of *f*_1_. The grey area represents *P*(*S_n_* ≤ 4.2), the probability of observing a sum of five RSCC values less than or equal to 4.2, which is given by integrating *f*_5_ from −∞ to 4.2, which is equivalent to evaluating the respective cumulative distribution function *F*(4.2) = 0.1426. The distribution of the random variable *S*_5_ peaks at 4.6 and *F*(4.6) = *P*(*S_n_* ≤ 4.6) = 0.71.

The wwPDB validation report contains two electron-density-related quality measures: a *Z*-score derived from the real-space *R*-value distribution (RSRZ) and the real-space correlation coefficient. We utilized the residue RSRZ value solely to compute the fraction of residues with RSRZ > 2, and refer to the caveats in using RSRZ as a validation criterion in §[Sec sec5]5. Since the RSRZ score is reported only for standard residues, but short peptide-ligand chains may contain nonstandard peptide residues, we took RSCC values to compute a continuous quality score for arbitrary chain lengths. To accomplish this, we first determined the N- and C-terminal residues of the peptide chain by locally aligning the amino-acid sequence derived from the PDB SEQRES record with the sequence implied by the ATOM/HETATM records of the respective chain using an alignment tool that allows arbitrary alphabets (https://github.com/eseraygun/python-alignment). The alignment step became necessary because the PDB SEQRES record lists the canonical chain of covalently bonded peptides, including those where three-dimensional data are missing. Conversely, the list of residues obtained from the ATOM/HETATM records also contains entities that do not belong to a peptide chain, for example atoms of buffer molecules. The alignment eliminated most nonpeptide groups and made sure that only residues with actual three-dimensional coordinate records entered the analysis. For a very few cases an alignment could not be generated successfully or we were not able to retrieve the validation report, reducing the number of available peptide ligand chains to 9747. We next determined the (empirical) probability density function (p.d.f.) *f*_1_ of 112 565 RSCC values extracted from these 9747 chains (Fig. 1 ▸ *a*).

In order to assess the overall quality of a peptide-ligand chain by the sum of RSCCs, we start with the assumption that these coefficients are independent of the position in the chain. Let *R* be a random variable that describes the event of observing a certain RSCC value in our data set, so *R* has p.d.f. *f*_1_. A peptide chain of length *n* (*n* > 1) is described by *n* independent and identically distributed random variables *R*_1_, *R*_2_, …, *R_n_* with p.d.f. *f*_1_. Let us begin with the case *n* = 2 and consider the sum of the two random variables *R*_1_ and *R*_2_. It is known that the p.d.f. of the random variable *S*_2_ = *R*_1_ + *R*_2_ is given by
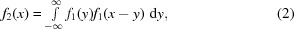
which is the convolution of *f*_1_ with itself. This allows us to define recursively
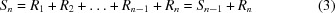
and compute its p.d.f. as
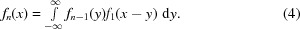
For any chain of length *n* with observed RSCC values *r*_1_, *r*_2_, …, *r_n_* corresponding to residues 1 to *n*, the probability of finding by chance the sum of RSCC values *s_n_* = *r*_1_ + *r*_2_ + … + *r_n_* or any lower sum is then given by *P*(*S_n_* ≤ *s_n_*). Fig. 1 ▸(*b*) illustrates the case *n* = 5 and provides numeric examples.

### 3.5. Overall score calculation   

The overall score was calculated as the sum of logarithms of normalized individual score ranks. We first computed the percentiles of the abovementioned bond-length, bond-angle, Ramachandran, side-chain rotamer and RSRZ outliers. During this step, missing measurements were replaced by the median of the respective distribution. This represents a very conservative approach, as all affected medians are zero (*i.e.* no outliers), except the fraction of RSRZ values greater than 2, where we computed a median of 1/21 = 0.047619 (corresponding to 4.76% RSRZ > 2 outliers per chain, *e.g.* one outlier in a chain of length 21). Notably, the medians remained identical before and after this replacement step. Furthermore, percentiles were also assigned to the *B*-factor statistics that contrast the *B* factors of peptide-ligand chain atoms with their neighbourhood atoms. The RSCC sum probabilities were left unchanged. The combined score was computed by a method used in gene prioritization, *MetaRanker* (Pers *et al.*, 2013 ▸), which has been reimplemented in the algorithm developed in Weichenberger *et al.* (2015 ▸). Briefly, each individual column is sorted such that the most interesting value ranks top, and its rank is then divided by number of all available scores for this column. For each entity to be scored, the logarithms of these normalized rank scores are then summed to derive the combined score. (We used the corresponding Dintor *Meta­Ranker* tool with default weights set to 1.) Sorting the results ascending by this score provides an ordered list of peptide-ligand chains with those most worthy of examination ranking top. More information on the keywords available for sorting our data and a detailed description of the variable names is provided in Supplementary Table S1.

### 3.6. Working with peptide *Twilight* data   

*Twilight* for small-molecule ligands includes a simple graphical user interface (GUI; Weichenberger *et al.*, 2013 ▸) that starts *Coot* (Emsley *et al.*, 2010 ▸) directly when clicking on a high-scoring ligand. With the increasing availability of reliable downloads from the PDB and in view of the necessity to examine larger stretches of electron density for peptides, we currently do not provide a GUI for the peptide data. The data table in tabular delimited format is available as Supporting Information.

We encourage readers to download our table and sort it as to their desire. It is possible to sort, for example, by authors or journal, and we leave this part of the analysis up to the curiosity of the user. To no surprise, some previously and publicly criticized structure models have been ‘top’ ranked (meaning identified as most problematic), and it seems that a disproportionate number of problem structure models come from the same groups or (corresponding) authors. This would indicate that certain schools propagate questionable practices, while the remainder perhaps can be classified as random aberrations. In any case, we stress again that the *Twilight* score alone is no indictment of a poor model. Instead, the actual electron density and the context and objectives of each publication need to be considered before judgement is rendered. Similarly to the small-molecule ligand examination, we emphasize that our findings and annotations are limited to the critique of *crystallographic* evidence for any given hypothesis. Other evidence may or may not exist that justifies a proposed biological hypotheses. As always, the provided annotations are our interpretation of the evidence, and naturally, in view of spurious or ambiguous density, interpretations may differ. However, in accordance with accepted scientific epistemology, strong evidence – as required in support of a strong claim such as a specific pose of a peptide ligand – generally does *not* lead to ambiguous interpretations.

### 3.7. Electron-density map contouring   

If not stated otherwise, figures were rendered with *PyMOL* (DeLano, 2008 ▸). The 2*mF*_o_ − *DF*_c_ maximum-likelihood maps are contoured at 1σ (blue) and *mF*_o_ − *DF*_c_ maps contoured at ±3σ (green/red) were calculated by *BUSTER-TNT* (Blanc *et al.*, 2004 ▸) after refinement of the model with the peptide omitted.

## 4. Problematic peptide ligands   

In our earlier analysis of small-molecule ligands, we classified problematic cases as detected by *Twilight* into groups based on visual inspection of the electron density (Pozharski *et al.*, 2013 ▸). Some of these classes are not appropriate when characterizing peptide ligands. Hence, we have evaluated over a hundred of the top protein–peptide complex structures detected by *Twilight* and provide a brief explanation and examples of the most distinct classes. Any such classification is inevitably subjective, and different scoring algorithms or a more stringent analysis may produce different results. Again, we stress that this analysis is based strictly on crystallographic evidence, and some additional caveats are expressed in §[Sec sec5]5.

### 4.1. Peptide ligands entirely unjustified by electron density   

In 23% of the examined top hundred cases no continuous electron density is observed to support the hypothesis that a peptide molecule is present in the crystal structure. Given that such purported peptide molecules are more often than not located near the protein surface, some superposed electron density tends to exist that clearly originates from water molecules. Very frequently, these peptide models are also characterized by rather implausible stereochemistry. A paradigmatic case involving antibody–peptide complexes has been commented on recently (Stanfield *et al.*, 2016*a* ▸,*b* ▸; Salunke *et al.*, 2016*a* ▸,*b* ▸; Fink, 2016 ▸), and the respective *Twilight* scores for PDB entries 2xzq, 2y06, 2y07, 2y36 (Khan & Salunke, 2012 ▸), 4bh7, 4bh8 (Khan & Salunke, 2014 ▸) and 4h0h (Tapryal *et al.*, 2013 ▸) cluster prominently at the top of the scores (ranking 12, 117, 92, 5, 353, 9 and 10, respectively, out of 9747 examined peptide complexes).

#### 4.1.1. PDB entries 3jti and 2pq2   

**Figure 2:**
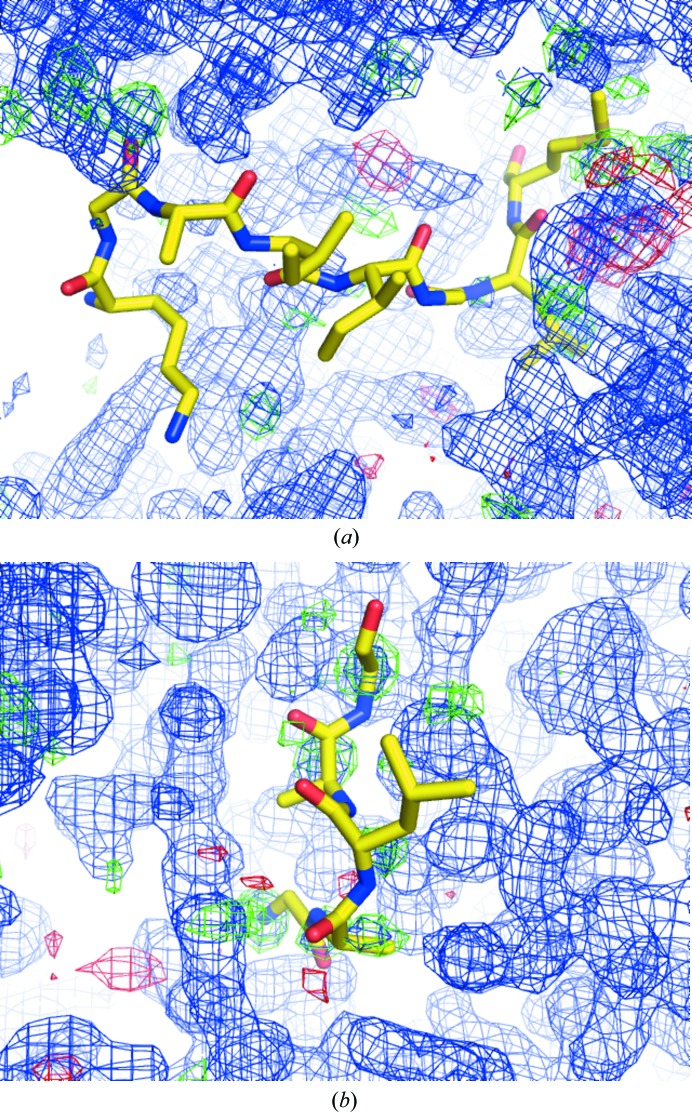
Examples of peptide molecules that do not appear to be justified by the electron density. Shown are electron-density maps for (*a*) the *Crystal structure of the complex formed between phospholipase A_2_ with β-amyloid fragment* (PDB entry 3jti) and (*b*) the *Structure of serine proteinase K complex with a highly flexible hydrophobic peptide* (PDB entry 2pq2), both at 1.8 Å resolution. Map parameters and contour levels for all figures are provided in §[Sec sec3.7]3.7.

These PDB entries illustrate the combination of poor evidence and vanishingly small prior probability, PDB entries 3jti (rank 7) and 2pq2 (rank 17) were selected from a prominently scoring cluster (Fig. 2 ▸). In addition to no evidence of electron density, as per the PDB report, the stereochemistry of the peptides is in the zeroth percentile for backbone torsions and side-chain conformers. Difference electron density indicates that the entire binding site in 3jti is poorly modelled. While the structures have remained ‘to be published’ since 2007 and 2009 with no associated publication record, they exemplify the problem of database contamination: with no associated publications that can be commented on, there is presently no clearly outlined procedure to remove such models from the PDB, short of per the author’s request. Policies on purging such problematic and misleading entries from the public record of structural models need to be established (Rupp *et al.*, 2016 ▸).

#### 4.1.2. PDB entry 2fys   

**Figure 3:**
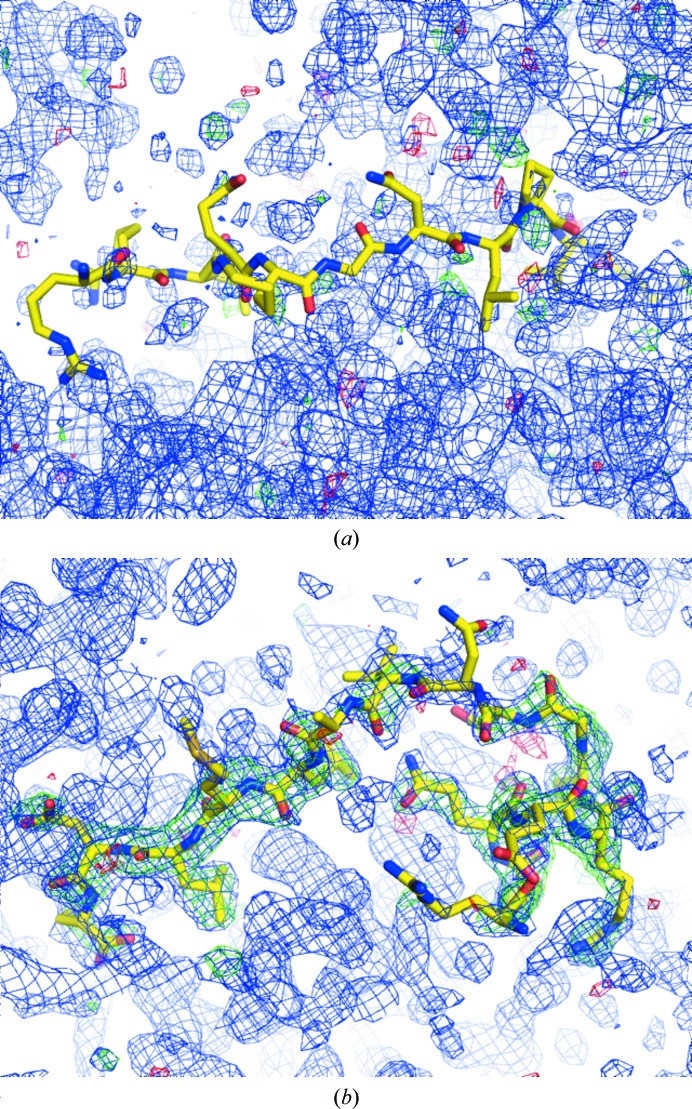
Evidence of the evident overinterpretation of the crystal structure of Erk2 in complex with ‘D-docking site peptides’. While the difference OMIT map for PDB entry 2gph (1.9 Å resolution) (*b*) confirms the presence and pose of the peptide, no such evidence is obvious for PDB entry 2fys chain *D* (2.5 Å resolution) (*a*).

To contrast the poor quality of models not even in the top ranks (132 for PDB entry 2fys chain *D* and 154 for PDB entry 2fys chain *C*) with models based on evidence as positive controls, we present two structures of the mitogen-activated protein kinase Erk2 in complex with a peptide from one of its many protein targets, MAP kinase phosphatase 3. Structures of this protein in complex with peptides aim to elucidate the molecular mechanism of this diverse target recognition. Analysis shows that there is little evidence in the difference electron-density map calculated for PDB entry 2fys with the peptide omitted (Liu *et al.*, 2006 ▸; Fig. 3 ▸ *a*), while for PDB entry 2gph (rank 3917; Zhou *et al.*, 2006 ▸) such evidence is unequivocal (Fig. 3 ▸ *b*).

### 4.2. Structures that contain significant disorder   

**Figure 4:**
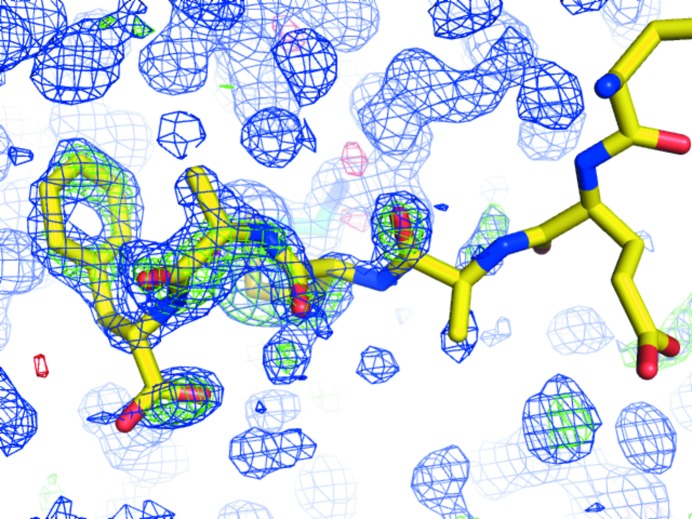
An example of a disordered peptide molecule. Shown are electron-density maps from the 1.38 Å resolution structure of bovine lactoferrin (PDB entry 3tod; *Twilight* rank 41; *Crystal structure of C-lobe of bovine lactoferrin complexed with 1-butyl-1*H*-pyrazole-5-carboxylic acid at 1.38 Å resolution*). Some electron density is clearly present that fits part of the peptide molecule well, while the rest of it is missing owing to disorder or other unknown reasons.

This category of 40% of examined structures contains two subclasses. (i) Electron density may show only the trace of the peptide, *e.g.* tubular density characteristic of the protein backbone, with little evidence of side chains. This may result in low RSCC values, despite the fact that the peptide molecule is likely to be present in the structure, but the exact sequence of register cannot be inferred from the crystallographic data. (ii) Clear electron density may be present for a well ordered part of the peptide, but the density becomes increasingly degraded towards the termini (Fig. 4 ▸). In these cases, it appears that the authors of the respective structures have included a longer peptide stretch than can be reasonably supported by evidence, at the cost of having to accept low RSCC values. While in both of these scenarios there is sufficient evidence at least for the presence of the peptide, the specific modelled conformation for the *entire* peptide cannot be fully supported. Nonetheless, the fact that the missing part of the peptide necessarily has to be in proximity to the modelled parts, excluding the space for other moieties, can be of value. While some suggestions have been made how to address the situation (Naschberger *et al.*, 2016 ▸; Kantardjieff *et al.*, 2002 ▸), there is no consensus yet as to how to include such parts of a molecule in a model.

### 4.3. Peptide ligands placed into electron density likely originating from mother-liquor components   

In 17% of the examined cases with density presumed to originate from (partially) ordered solvent components some ambiguity exists because we did not attempt to identify which buffer components might be contributing to the electron density. In most of these cases one or several blobs of electron density roughly the size of a glycerol molecule are located near the protein surface. Several examples of buffer or crystallization-cocktail molecules mistakenly identified as the ligands of desire are illustrated in Pozharski *et al.* (2013 ▸). Tubular density frequently results from partly ordered PEG molecules, and care must be taken not to overinterpret such density as the presence of a peptide. Particularly at lower resolution, the electron density of PEGs resembles the trace of a peptide backbone. Evaluating the chemical plausibility of the interaction patterns of the peptide ligand with the protein may help in these instances (see Naschberger *et al.*, 2016 ▸).

### 4.4. Peptide ligands extended by noncrystallographic symmetry   

**Figure 5:**
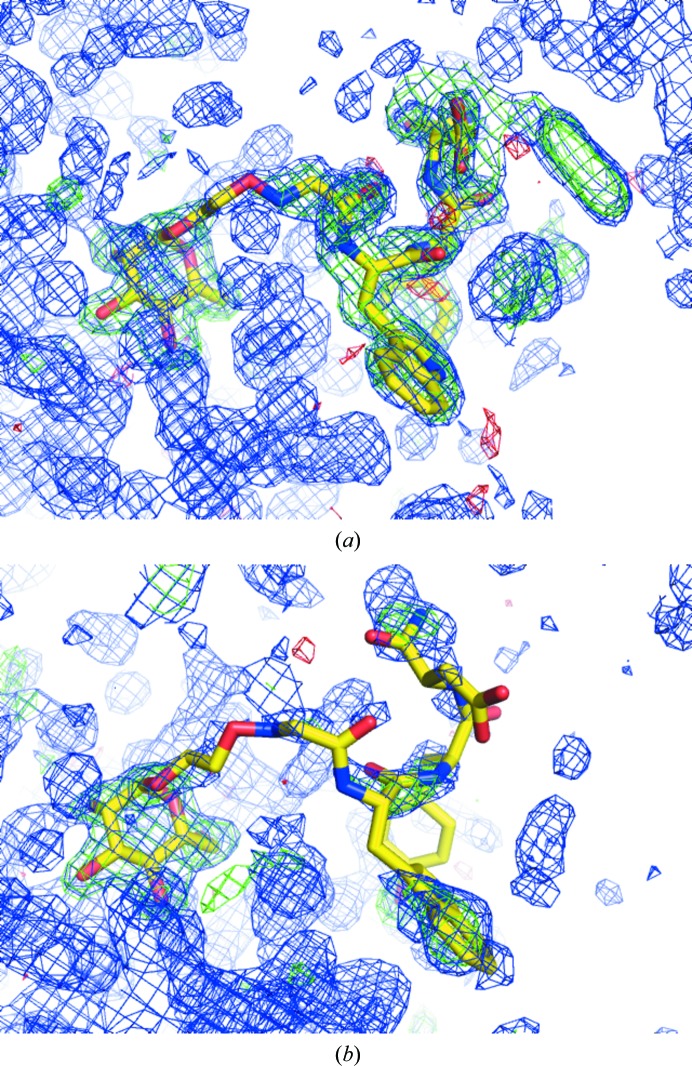
A peptide molecule that appears to be projected from the binding site of a noncrystallographic symmetry (NCS)-related copy. Shown are electron-density maps from the 1.9 Å resolution structure of concanavalin A in complex with a glycomimetic peptide (PDB entry 4czs). Two peptide molecules (chains *E* and *F*; ranks 3105 and 2528) are well traceable in electron density [chain *E* is shown in (*a*)], while chains *G* and *H* (rank 254 and 99, respectively) are only partly visible in electron density [chain *H* is shown in (*b*)] (Ng *et al.*, 2015 ▸). The original publication shows only a surface rendering with a ball-and-stick model of the glycopeptide (unspecified chain ID, without electron density).

Several complex structures (11% of the examined models) included multiple noncrystallographic symmetry (NCS)-related copies of both the protein and the peptide. In some of the copies the electron density for the peptide was very clear and the particular chain was not flagged by *Twilight* analysis as suspect. In contrast, other chains in the same molecule were clearly flagged. It is possible that authors place peptide molecules into each binding site of multiple copies of the protein in the crystal structures, even when the electron-density evidence for some of the NCS-related binding sites is rather weak. Similar observations are also true for small-molecule ligands and, as a matter of good practice, the temptation to present the best electron density as ‘representative’ when other NCS-related binding sites are much less convincing should be resisted. Ignoring the less convincing instance indicates cognitive bias: omission of negative results is known as confirmation bias. NCS-related sites are crystallographically *not* equivalent: different site accessibility or plasticity of the binding site can be valid – and actually quite interesting – reasons for unequal site occupancies. An example of different peptide occupancies in NCS-related copies is provided in Fig. 5 ▸.

### 4.5. Incorrectly modelled peptides   

In 2% of the examined models, while electron density is present and appears to resemble the proposed peptide, the latter is not correctly placed. Such models can in fact be corrected and could benefit from manual or automated re-refinement, for example *via PDB_REDO* (Joosten *et al.*, 2011 ▸). In such instances, deposited diffraction data prove their value as a basis for improved ligand models. Unfortunately, in the disturbing situation of ligands modelled into empty space or noise density, no improvement of the ligand is possible. Nonetheless, a more plausible model of the entire crystal structure, *sans* ligand, can probably be refined.

### 4.6. Incorrectly scored peptide ligands   

In 6% of the cases, incorrect false positives and false negatives were scored. In the former case, a low RSCC was reported by the PDB for a peptide-ligand model that actually presents a good fit to the electron density. This includes obvious deposition problems (*e.g.* PDB entry 4yyj contains two peptide ligands, and for unknown reasons one is assigned extremely high *B* factors). False negatives (missed entries with poor density but acceptable stereochemistry) can result from that fact that EDS density is not ligand-OMIT density and therefore rather biases the density *in favour of the presence* of a ligand rather than its absence. Some weak 2*mF*_o_ − *DF*_c_ density at levels around 0.6σ in clean high-resolution EDS maps may in fact be the result of model bias.

**Figure 6:**
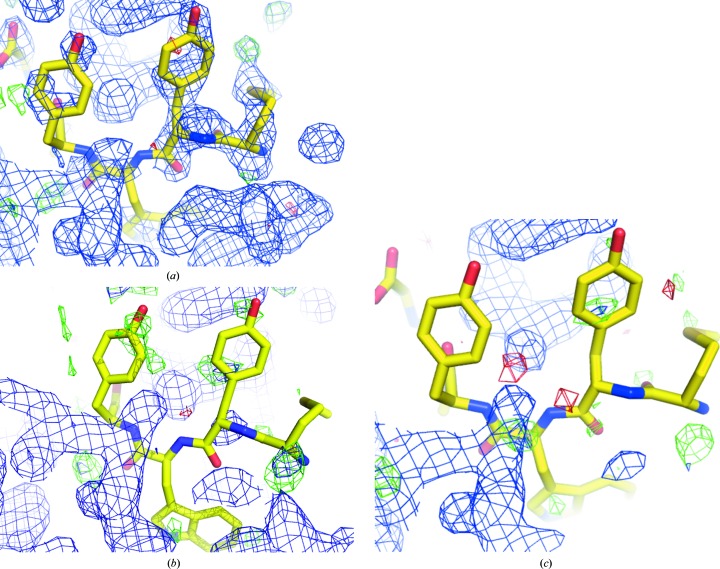
Evidence of model bias in electron-density maps produced by EDS. Shown are 1.93 Å resolution electron-density maps of the same region of the concanavalin A–peptide complex (PDB entry 1jw6; rank 116; Zhang *et al.*, 2001 ▸) as calculated by EDS (*a*), *PDB_REDO* (*b*) and by *BUSTER-TNT* after refinement of the model with the peptide molecule omitted (*c*). The peptide is shown in yellow; the protein model has been omitted for clarity.

It is important to emphasize that the *Twilight* electron-density fit classification is based on electron density produced by EDS, which is inherently model-biased. We are certain that at least in some cases biased non-OMIT electron density produces a more positive outlook of the peptide as being simply disordered rather than entirely unsupported by electron density. Comparison of the electron-density maps calculated directly from the deposited model and one produced after optimization by *PDB_REDO* (Figs. 6 ▸ *a* and 6 ▸ *b*) indicates that some peptide models are difficult to justify in the unbiased electron density. The presence of deceiving bias in non-OMIT maps becomes obvious when the peptide model is omitted from the model (Fig. 6 ▸ *c*), and illustrates that it is likely that some of our classification of the problematic peptides may in fact be *too optimistic* with regard to the degree to which their presence may be deduced from electron density alone.

### 4.7. Problems not reflected in *Twilight* scores   

In certain instances, the *Twilight* peptide score alone simply does not do justice to the bizarre nature of some models. The reader may examine PDB entry 3zg5 (ranks 1 and 115; Otero *et al.*, 2013 ▸), and the entire binding site around chains *C* and *D* which shows a number of cataclysmic steric clashes defying any prior knowledge. We assume that the PDB validation reports were available in 2013, and about 20 pages of steric clashes combined with an absence of reasonable density seem to have evaded the reviewers as well as the journal editors.

## 5. Caveats and recommendations   

### 5.1. One scientist’s garbage is another one’s ligand   

Any analysis based on unsupervised machine learning needs to be treated with caution, including our *Twilight*. Just as reviewers (should) do, we rely on publicly available validation records produced by the PDB, or use the same programs as the PDB, such as *MolProbity* (Chen *et al.*, 2010 ▸), to obtain geometry-based statistics. The validation programs must cover all instances of depositions, and neither they nor we are aware of the context in which the structure model was referenced or used to support a claim. In clear-cut cases, where a combination of lacking evidence and vanishing prior probability puts a model squarely into the top ranks of vanishing posterior probability, this is not a concern. However, even weak posterior probability of a model may serve some purpose in a given context. Weak or incomplete density of a molecule with reasonable stereochemistry can lend partial support to accompanying and substantial biochemical evidence for a specific hypothesis or proposal. The key term here is *partial* support.

### 5.2. Database contamination hampers data mining   

It is difficult to draw the line between useless models and acceptable ones. While the latter may be a subjective and specific matter, policies on purging clearly problematic and misleading entries from the public record of structural models need to be established. Users rely on public data repositories for data mining and wrong entries produce erroneous results, as exemplified in the comment of Raczynska *et al.* (2016 ▸) on the meta analysis of Zn^2+^-binding sites by Yao *et al.* (2015 ▸). Additional examples of problems caused by wrong or inconsistent database entries are highlighted in Dauter *et al.* (2014 ▸) and Minor *et al.* (2016 ▸).

### 5.3. Preserving the integrity of the record   

The present policy of the PDB that the depositing author has to agree to the removal (obsoleting) of a PDB entry is not sufficient nor can it be consistently applied. It has been overridden in the recent retraction (Abdul Ajees *et al.*, 2016 ▸) by the journal of a fabricated structure published a decade ago (Abdul Ajees *et al.*, 2006 ▸), where the depositing author explicitly did not agree with the retraction. This case also sets a precedent that the journal editors assume a large degree of responsibility for keeping the record straight. Unfortunately, this responsibility seems to be rarely executed when it comes to assuring adequate review. The burden is generally left to critical voices who take the effort to contribute dissenting comments that are mostly ignored (Rupp *et al.*, 2016 ▸). Even when the absence of evidence is acknowledged (Fink, 2016 ▸), the editors shy away from demanding a retraction and the PDB from obsoleting problematic models. A deep-rooted problem seems to be a resistance to the correction of errors, as documented by the persistence of false positives in the scientific literature (Simmons *et al.*, 2011 ▸). Once a finding has passed review and is in print (or in the PDB), it becomes very hard to correct. The ensuing problems of database contamination and the persistence of publications based on incorrect structure models must be effectively addressed by the entire structural biology community.

### 5.4. Evading error detection   

No validation program can anticipate each and every situation where certain practices may – not even on purpose – help to evade validation software. For example, a very simple procedure to gain credibility for a ‘no-density’ peptide model based on prior probability would be (i) to restrain the backbone torsion angles or (ii) not to refine, but simply place, a built peptide model with good initial stereochemistry into the binding site. Both cases could probably be detected by re-refinement using *PDB_REDO* (Joosten *et al.*, 2009 ▸, 2011 ▸) prior to validation.

### 5.5. When parsimony cuts short   

The epistemology of empirical science also agrees that a model should be parsimonious; that is, not include more parameters than necessary. Biomolecular crystallographers treat this concept flexibly, and no consensus exists regarding to what levels weak density should be interpreted. One almost always suffers a penalty for modelling into very weak density by receiving low real-space correlation as a result of a reduced certainty in where to place the atoms. The weak scattering contributions in turn leave the refinement program relying almost solely on restraints, which are not sufficient to restore reasonable geometry, particularly in the case of side-chain torsions or generally unrestrained backbone torsions. Excessive Ramachandran outliers in poorly defined target protein loops or ligand peptide stretches are the frequent result.

While superficially in the spirit of parsimony, modelling and depositing only a few residues of an entire peptide achieves unsuspicious statistics, and prevents in essence the scenario depicted in Fig. 4 ▸. Evaluation of the validity of such models (not from a crystallographic viewpoint but as support for a hypothesis) does require careful reading of the entire manuscript beyond map and geometry evaluation. As a very recent example that has passed our analysis we offer PDB entry 5hdt (Ouyang *et al.*, 2016 ▸), which did not receive a concerning score (rank 1232 out of 9747 scored models) owing to acceptable real-space correlation and reasonable stereochemistry. However, the presence of an entire 33-residue peptide is inferred from only five residues present in the model (1–6 and 12–33 are missing; Ouyang *et al.*, 2016 ▸). While biological evidence may well indicate that the entire peptide is bound, purely crystallographic evidence for its entirety is not given (and, technically correct, also not modelled).

### 5.6. Modelling with restraint has its rewards   

The fact that many bound peptides appear disordered and not traceable in electron density at their termini also causes a high percentage of RSRZ > 2 outliers. In regular proteins, the percentage of poorly modelled residues is generally a few percent only, but in short bound peptides the percentage can also reach much higher numbers in otherwise well modelled peptides. For example, a five-residue peptide with one poorly modelled terminal residue would score 20% RSRZ outliers, which would be unacceptable for a protein target, but could have little effect on the interpretation of the peptide binding mode. The general advice then would be to exercise reasonable restraint in modelling and model only what has reasonable support in the form of electron density. In this case, the scientist himself, and not acceptable RSRZ statistics, must then explain why modelling for example only five out of 33 peptide residues allows an unambiguous assignment of the peptide residues specified in target protein binding.

### 5.7. Training and teaching   

While most of the peptide-ligand models are reasonably justified by evidence and prior expectations, a number of very poor cases appear in the top ranks. A strong claim of a peptide bound in a specific pose does require equally strong evidence, which for the high-ranking models is often simply absent. In this context, the necessity to provide properly reconstructed positive OMIT electron density of the ligand as a proof positive for the claimed ligand pose should be emphasized.

Even more disturbing than absence of evidence is the fact that most ‘top-ranking’ peptides also fail the test of prior probability, meaning that many are in the zeroth percentiles of expected stereochemistry, indicating high-energy, strained conformations for which even stronger experimental evidence would be necessary. Needless to say, this violation of the most basic stereochemistry known to every student of structural biology sheds a disturbing light on the presentation of such an improbable model without evidence.

Some of the cases of problematic models may be attributable to a young researcher directed to perform protein crystallo­graphy without receiving adequate training and expert advice. Such findings do not instill much confidence in the quality of the training that the students and post-doctoral researchers receive there. The commoditization of protein crystallography and the easy access to powerful synchrotron resources and almost failsafe software on one hand allow teachers to focus less on mathematical and technical training in crystallography, but there is certainly an unmet need to (re)train students in the basics of scientific epistemology. Biomolecular crystallography, with its strong foundation in Bayesian reasoning and inference, seems to be an excellent place to start. Despite all the diagnostics and validation tools available, one needs to realise that not the validation statisticians, but the individual crystallographer, bears the final responsibility for the correctness of the deposited model.

## Supplementary Material

Supporting Information: Raw data in delimited format.. DOI: 10.1107/S205979831601620X/ba5251sup1.txt

Supporting Information: Supplementary Table S1.. DOI: 10.1107/S205979831601620X/ba5251sup2.pdf
