# Supplementary material for: Twilight reloaded: the peptide experience
Source: Acta Crystallogr D Struct Biol. 2017 Feb 28;73(Pt 3):211–22. doi: 10.1107/S205979831601620X (PMC5349433; doi:10.1107/S205979831601620X)
Supplement: Supplementary file 2 [file d-73-00211-sup2.pdf]

STRUCTURAL
BIOLOGY

Volume 72 (2016)

Supporting information for article:

Twilight reloaded: the peptide experience

Christian X. Weichenberger, Edwin Pozharski and Bernhard Rupp

Table S1 Description of the column names in the Twilight peptide ligand table.

**Column name**	**Description**
key	Unique key formed by concatenation of PDB identifier and chain name.
PDBID[#]	PDB entry four letter code.
Chn[#]	Chain name. All parameters listed in this table starting from “Ratio” refer to this chain.
DepDate[#]	Deposition date.
Resol[#]	Experimental resolution (Å).
Robs[#,&]	Observed-set (working set and test set taken together) *R* value.
Rwork[#,&]	Working-set *R* value. A negative number indicates its absence.
Rfree[#,&]	Test-set *R* value (R_{free}). A negative number indicates its absence.
Authors[#]	Comma-separated list of authors.
Jrnl[#]	Abbreviated journal name if an article describing the structure has been published. Contains ‘To be Published’ if no publication is associated with the PDB entry.
PMID[#]	NCBI PubMed identifier of the primary citation, if the structure has been published; otherwise, the cell contains the hyphen character ‘-’.
ChLen[#]	Number of residues according to PDB SEQRES record.
Ratio	Ratio between the number of residues of chain “Chn” and the longest chain present in entry “PDBID”.
RSCCSm	Sum of all available RSCC absolute values for chain “Chn”.
NrRSCC	Number of residues furnished with an RSCC value.
FracRSRZgt2[*,D]	Number of residues with an RSRZ value greater than 2.0 divided by the overall number of residues with an RSRZ value.
RamaFav[*]	Number of residues contained in the ‘favored’ region of MolProbity’s Ramachandran plot divided by the number of residues with Ramachandran angles available.
RamaAlwd[*]	Same measure as “RamaFav”, only with residues in ‘allowed’ region.
RamaOut[*,D]	Same measure as “RamaFav”, only with residues in neither ‘allowed’ nor ‘favored’ region.
RotaAlwd[*]	Number of side-chains with rotameric conformation normalized by the number of all residues with rotamer information.
RotaOut[*,D]	Number of side-chains with non-rotameric conformation normalized by the number of all residues with rotamer information.
BondLenOut[*,D]	Number of bond lengths differing by more than 5 standard deviations from the expected value divided by the number of all bond lengths observed.
BondAngleOut[*,D]	Number of bond angles differing by more than 5 standard deviations from the expected value divided by the number of all bond angles observed.
ptlFracRSRZgt2[*,D]	Percentiles of the variables described by columns “FracRSRZgt2”, “RamaOut”, “RotaOut”, “BondLenOut”, “BondAngleOut”. Values are normalized to be between 0 and 1, e.g. the 95th percentile is written as 0.95.
ptlRamaOut[*,D]	
ptlRotaOut[*,D]	
ptlBondLenOut[*,D]	
ptlBondAngleOut[*,D]	
ProbRSCCSm[A]	Probability to observe an RSCC sum of “RSCCSm” or higher given “NrRSCC” residues.
bs[D]	B-factor statistics calculated as the difference of the average B-factor of atoms belonging to “Chn” and atoms within 6 Å of any atom from “Chn”.
ptlbs[D]	Percentile for the variable in column “bs”.
Rank Score (ptlFracRSRZgt2, w=1.00) [*,A]	Rank score computed by Dintor’s MetaRanker tool. Respective column names are indicated in parentheses, followed by the weight given to the rank score in the overall score. (All weights default to 1.)
Rank Score (ptlRamaOut, w=1.00) [*,A]	
Rank Score (ptlRotaOut, w=1.00) [*,A]	
Rank Score (ptlBondLenOut, w=1.00) [*,A]	
Rank Score (ptlBondAngleOut, w=1.00) [*,A]	
Rank Score (ProbRSCCSm, w=1.00) [A]	
Rank Score (ptlbs, w=1.00) [A]	
Meta Rank Score (key) [A]	Overall MetaRanker score for PDB entry “PDBID” and chain “Chn”. The table is sorted ascending by this score.

[#]Value directly taken from the result list returned by the PDB Advanced Search Interface query.

[&]A negative number indicates its absence.

[*]An “NA” entry indicates a missing value.

[A]Ascending order places lowest quality chain on top.

[D]Descending order places lowest quality chain on top.
